# E2F1-Mediated *FOS* Induction in Arsenic Trioxide–Induced Cellular Transformation: Effects of Global H3K9 Hypoacetylation and Promoter-Specific Hyperacetylation *in Vitro*

**DOI:** 10.1289/ehp.1408302

**Published:** 2015-01-09

**Authors:** Sunniyat Rahman, Zjwan Housein, Aleksandra Dabrowska, Maria Dolores Mayán, Alan R. Boobis, Nabil Hajji

**Affiliations:** 1 Centre for Pharmacology and Therapeutics, Department of Medicine, Imperial College London, London, United Kingdom; 2 Osteoarticular and Aging Research Group, Rheumatology Division, INIBIC-Hospital Universitario, Coruña, Spain

## Abstract

Background: Aberrant histone acetylation has been observed in carcinogenesis and cellular transformation associated with arsenic exposure; however, the molecular mechanisms and cellular outcomes of such changes are poorly understood.

Objective: We investigated the impact of tolerated and toxic arsenic trioxide (As_2_O_3_) exposure in human embryonic kidney (HEK293T) and urothelial (UROtsa) cells to characterize the alterations in histone acetylation and gene expression as well as the implications for cellular transformation.

Methods: Tolerated and toxic exposures of As_2_O_3_ were identified by measurement of cell death, mitochondrial function, cellular proliferation, and anchorage-independent growth. Histone extraction, the MNase sensitivity assay, and immunoblotting were used to assess global histone acetylation levels, and gene promoter-specific interactions were measured by chromatin immunoprecipitation followed by reverse-transcriptase polymerase chain reaction.

Results: Tolerated and toxic dosages, respectively, were defined as 0.5 μM and 2.5 μM As_2_O_3_ in HEK293T cells and 1 μM and 5 μM As_2_O_3_ in UROtsa cells. Global hypoacetylation of H3K9 at 72 hr was observed in UROtsa cells following tolerated and toxic exposure. In both cell lines, tolerated exposure alone led to H3K9 hyperacetylation and E2F1 binding at the *FOS* promoter, which remained elevated after 72 hr, contrary to global H3K9 hypoacetylation. Thus, promoter-specific H3K9 acetylation is a better predictor of cellular transformation than are global histone acetylation patterns. Tolerated exposure resulted in an increased expression of the proto-oncogenes *FOS* and *JUN* in both cell lines at 72 hr.

Conclusion: Global H3K9 hypoacetylation and promoter-specific hyperacetylation facilitate E2F1-mediated *FOS* induction in As_2_O_3_-induced cellular transformation.

Citation: Rahman S, Housein Z, Dabrowska A, Mayán MD, Boobis AR, Hajji N. 2015. E2F1-mediated *FOS* induction in arsenic trioxide–induced cellular transformation: effects of global H3K9 hypoacetylation and promoter-specific hyperacetylation *in vitro*. Environ Health Perspect 123:484–492; http://dx.doi.org/10.1289/ehp.1408302

## Introduction

Arsenic (As) is regarded as one of the most toxic naturally occurring carcinogenic metalloids (Chen et al. 1985; Hajji et al. 2010). Arsenic-containing substances can be broadly classified into two groups, organic and inorganic arsenicals, with the latter being considered to be more toxic to human health. Inorganic species such as arsenite (As^III^) and arsenate (As^V^) are ingested primarily through contaminated drinking water and can lead to negative health outcomes (Grund et al. 2008). The acute dose for arsenic poisoning (0.17–0.87 mg/kg body weight) can result in diarrhea and vomiting as well as kidney and liver toxicity (Yu et al. 2006). Long-term exposure to arsenic can lead to severe detrimental health outcomes including cardiovascular disease and malignancies of the skin, lung, and bladder (Kapaj et al. 2006; Nelson et al. 2006). Although epidemiological studies have extensively characterized the association between chronic arsenic exposure and the rising incidence of malignant-based mortality, a mechanistic understanding of arsenic-induced carcinogenicity remains to be established (Kurttio et al. 1999). Proposed mechanisms include the generation of reactive oxygen species, cytotoxicity due to the formation of a reactive metabolite, inhibition of DNA repair, chromosomal aberrations, uncontrolled cellular proliferation, and altered DNA methylation patterns (Shi et al. 2004).

A mounting body of evidence suggests that epigenetic mechanisms may play a role in the carcinogenicity of arsenic, not only through aberrant DNA methylation but also through altered expression of microRNAs and changes in histone modifications (Ren et al. 2011). Alterations in DNA methylation in response to arsenic exposure is relatively well documented, from the identification of arsenic as a repressor of DNA methyltransferase expression, to the detection of a number of hypo- and hypermethylated tumor-suppressor and oncogenic promoter loci such as *p15*, *p16*, *p53*, *Hras1*, *Myc*, and *Esr1* (Chanda 2006; Reichard and Puga 2010). Increased global DNA methylation of peripheral blood mononuclear cells has also been observed and correlated with water, urinary, and blood arsenic levels in Bangladeshi adults (Niedzwiecki et al. 2013). Beyond DNA methylation, little is known about the effects arsenic has on the higher-order chromatin structure. An increasing body of evidence suggests that posttranslational histone modifications, particularly histone acetylation, can influence overall chromatin structure and gene transcription, with clear functional consequences in cellular processes such as proliferation and apoptosis (Füllgrabe et al. 2010). Suitably, alterations of histone modification as a result of arsenic exposure have been identified, particularly changes in phosphorylation, methylation, and acetylation; however, relating such modifications to a mechanistic outcome has been limited (Jo et al. 2009; Li et al. 2002; Zhou et al. 2008).

Arsenic exposure has been shown to increase global histone acetylation via the inhibition of histone deacetylases (HDACs) at an intensity comparable to the HDAC inhibitor triochostatin A (Iacobuzio-Donahue 2009). Two functionally antagonistic enzyme groups mediate histone acetylation, comprising three major families of histone acetyltransferases (HATs) and four classes of HDACs. Because of the opposing functional nature of the HDACs and HATs, Peserico and Simone (2011) suggested that the relative balance, either through expression or activity, alters the physiology of cells and provides an insight into the pathological outcomes in a number of diseases, including cancer. This equilibrium needs further study in the context of arsenic exposure.

Although multiple arsenic species have been used in carcinogenesis studies, the use of low-dose arsenic trioxide (As_2_O_3_) exposure has proven to be suitable in studies pertinent to cellular transformation and epigenetic aberrations. Low-dose (0.01–1 μM) As_2_O_3_ exposure for 24 hr has been shown to increase proliferation and cell cycle progression in normal breast epithelial cells (MCF10A) via the elevated expression of CDC6 and cyclin D1 (Liu et al. 2010). Kim et al. (2012) reported that exposure of BALB/c 3T3 cells to tolerated doses of As_2_O_3_ led to not only cellular transformation and tumor generation in nude mice but also to a time-dependent increase in H3K27 trimethylation mediated by the polycomb proteins BM1 and SUZ12. Exposure of lung epithelial BEAS-2B cells to a tolerated dose of As_2_O_3_ resulted in an increase in cellular proliferation and the differential expression of genes regulating histone H1, H3, and H4, as determined by an *in silico* pathway analysis (Stueckle et al. 2012). Additional research is required to investigate the carcinogenic mechanisms of As_2_O_3_, because human exposure to this species of arsenic is not limited to tolerated environmental exposures, but also can occur through cytotoxic anticancer therapies (Soignet et al. 1998). Thus, comparisons between tolerated and toxic exposures of As_2_O_3_ require further examination.

In this study we characterized the tolerated and toxic profile of As_2_O_3_ exposure by analyzing multiple cellular survival parameters. We defined a tolerated exposure as one that induced significant biological effects without causing toxicity, and a toxic exposure as one that induced significant biological effects leading to toxicity and cell death. Identification of a tolerated concentration ensured epigenetic characterization of the relevant cellular context, that is, continued proliferation and cellular tolerance rather than toxicity. We also characterized the higher-order chromatin conformation and histone acetylation changes that occur after exposure to As_2_O_3_ at tolerated and toxic dosages and those that occur between early and more prolonged exposure. We explored the impact of As_2_O_3_ exposure on HDAC and HAT expression and determined whether specific alterations in histone acetylation impinge on known proto-oncogenic promoters, altering gene expression and leading to increased proliferation and carcinogenic potential.

## Materials and Methods

*Cell culture*. Human embryonic kidney (HEK) 293T cells and human urothelial (UROtsa) cells were generously donated by Bertrand Joseph (Karolinska Institutet) and Scott Garrett (University of North Dakota), respectively. Cells were grown in T75 flasks (Sarstedt) with complete medium [Dulbecco’s modified Eagle’s medium (DMEM; Sigma) completed with 10% fetal bovine serum (Sigma), 1% l-glutamine (Gibco), and 1% penicillin–streptomycin solution (Sigma)]. All cells were cultured at 37°C in a humid 5% CO_2_ atmosphere.

*Two-step quantitative real-time reverse-transcriptase polymerase chain reaction (qRT-PCR)*. Total RNA was extracted using the RNeasy Mini Kit (Qiagen) according to the manufacturer’s protocol. For two-step qRT-PCR, we first synthesized cDNA using the ThermoScript RT-PCR System (Invitrogen) with a 5-μg input RNA amount for each sample. qRT-PCR was then carried out on an ABI7500 Fast Real-Time PCR System (Applied Biosystems) with a Platinum SYBR Green qPCR SuperMix-UDG (Invitrogen) used according to the manufacturer’s protocol. For quantification, we used the standard curve method described in detail by Bookout et al. (2006). The primers used are provided in Supplemental Material, Table S1.

*Clonogenic assay*. For the clonogenic assay, HEK293T cells were seeded into six-well dishes at 500 cells/well and incubated overnight in complete medium. The next day the medium was changed and the As_2_O_3_ was added (concentrations of 0.5–2.5 μM), with no further changes of medium or replenishment. After the exposure period, cells were washed with phosphate-buffered saline (PBS) and fixed in 100% methanol for 10 min at –20°C. Colonies were stained with a solution of 5% Giemsa, 25% methanol, and 70% PBS and then counted. The clonogenic assay has been described in detail (Hajji et al. 2010).

*Proliferation assay*. To examine proliferation, cells were seeded into 96-well plates at 4 × 10^3^ cells/well and incubated overnight in 100 μL of complete DMEM. Cells were then incubated for 72 hr with As_2_O_3_ in 100 μL complete DMEM at final concentrations of 0.5 and 2.5 μM. After treatment, 10 μL of Cell Proliferation Reagent WST-1 (Roche) was added to each well and the plates were incubated for 4 hr at 37°C and 5% CO_2_. Medium was homogenized on a plate shaker for 1 min, and absorbance was then measured at 440 nm in a plate spectrophotometer (FLUOstar OPTIMA; BMG Labtech). Absorbance readings were subtracted from a background measurement at 440 nm, which is the absorbance of culture medium with WST-1 in the absence of cells. Data are presented as a proliferative index in arbitrary units.

*Anchorage-independent growth*. Soft agar plates were prepared using two agar densities. The base layer contained 0.8% Noble Agar (Sigma) dissolved in H_2_O and supplemented with DMEM (Sigma) at a 1:1 ratio. Cells pretreated with As_2_O_3_ as described above were plated at a density of 2.5 × 10^3^ in a 0.4% Noble Agar top layer, and supplemented DMEM was replenished every 3 days. After 14 days, plates were stained with 0.005% Crystal Violet (Sigma) and 20% methanol (Sigma) diluted in 1× PBS (Gibco) for 2 hr. Colonies were examined under a stereomicroscope. Details regarding this method have been described by Raptis and Vultur (2001).

*Histone extraction*. For histone extraction, cells were disrupted on ice in a lysis buffer (10 mM Tris, pH 6.5, 50 mM sodium bisulphate, 10 mM magnesium chloride, 8.6% sucrose, 1% Triton X-100). Samples were centrifuged at 10,000 × *g* for 10 min at 4°C. Pellets were then washed in ice-cold Tris-EDTA (10 mM Tris, pH 7.4, 13 mM EDTA) and centrifuged. Histones were precipitated by acid extraction as previously described (Hajji et al. 2010).

*Western blots*. We detected acetylation of core histones and relative levels of intracellular cellular survival components by immunoblotting. We used the following antibodies: for histone acetylation analysis: anti-acetyl histone H3-Lys 9 (H3K9), anti-acetyl histone H4-Lys 12, and anti-acetyl histone H4-Lys 16 (all from Millipore); for intracellular protein analysis: anti-PARP, MDM2 (mouse double minute 2 homolog), p53 phosphorylated serine 15, p53, BID, caspase-3, and β-actin (all from Cell Signaling Technology). Cell lysates were separated by SDS-PAGE under reducing conditions and transferred to a nitrocellulose membrane (GE Life Science). Membranes were visualized using enhanced chemiluminescence (ECL) reagents and exposure to ECL hyperfilm (GE Healthcare). Blots were quantified by densitometric analysis using ImageJ software (National Institutes of Health; http://rsb.info.nih.gov/ij/), with data normalized against β-actin and total histone proteins to correct for loading. Antibodies used are listed in Supplemental Material, “Antibodies.”

*Protein extraction*. Total protein extraction was achieved by disrupting harvested cells with TGN buffer [1 M Tris-HCL, 2.5 M NaCl, glycerol, 0.5 M β-glycerphosphate, 1% Tween 20, and 0.2% Nonidet P40; completed with EDTA-free protease inhibitor (Roche)]. Samples were centrifuged at maximal speed for 10 min, yielding the proteins in the supernatant.

*Flow cytometry*. We identified mitochondrial superoxide anion reactive oxygen species (ROS) in live cells using MitoSOX Red flourogenic dye (Invitrogen) according to the manufacturer’s guidelines. Cells were harvested, washed with PBS, suspended in 250 μL of 2.5 mM Mitosox Red dissolved in PBS, and allowed to incubate for 20 min under cell culture conditions in the dark. Mitochondrial superoxide was measured by flow cytometer (FACSCalibur; BD Biosciences). MitoSOX Red was excited by laser at 488 nm and detected in the FL2 channel.

After cells were harvested and washed as described above, mitochondrial membrane potential (Δψ_m_) and apoptosis were assayed by incubating the cells in 80 μM 3,3´-dihexyloxacarbocyanine iodide and propidium iodide for 20 min at 37°C in the dark. The samples were then analyzed using a FACSCalibur flow cytometer. Cells were fixed with 70% ethanol and then stained with 50 μM propidium iodide (Sigma) for cell cycle analysis. Fluorescence was measured using the FL2 channel on a FACSCalibur flow cytometer. Data were analyzed using Cyflogic, version 1.2.1 (CyFlo Ltd.; http://www.cyflogic.com/).

*Poly (ADP-ribose) polymerase 1 (PARP) cleavage*. We used PARP cleavage, a hallmark of apoptosis (Duriez and Shah 1997), as an end point to identify toxicity induced by As_2_O_3_ exposures. PARP cleavage was evaluated by Western blotting as described above.

*Transfections*. We used the following plasmids for transfections: HDAC1 (plasmid 13820), HDAC3 (plasmid 13819), and HDAC4 (plasmid 13821; all from Addgene). HDAC2 was generously provided by Ito Kazuhiro (Imperial College London), and PCAF, HMOF, and TIP60 were kindly provided by Bertrand Joseph (Karolinska Institutet) (see Supplemental Material, “Plasmids”). Transient transfections were performed using filter-sterilized polyethylenimine (PEI) reagent (Polysciences) at 1 mg/mL in H_2_O (pH 6.8). Transfection reagent and plasmid complex were synthesized using a 1:4 (wt/vol) DNA to PEI ratio made up to 200 μL in DMEM with no supplements before drop-wise addition to HEK293T cells. After transfection, cells were allowed to rest for 24 hr in fresh DMEM and then treated with As_2_O_3_.

*Microccocal nuclease (MNase) sensitivity assay*. Cells were lysed in NP-40 lysis buffer, and nuclei were resuspended in MNase digestion buffer. A total of 0.025 units of MNase (N3755; Sigma) was added to each sample, and samples were incubated for 5 min at 15–20°C. The reaction was stopped by the addition of MNase digestion buffer, MNase stop buffer, proteinase K, and 20% SDS followed by overnight incubation. DNA was extracted by standard phenol/chloroform extraction and ethanol precipitation. Detailed methods and reagents have been described previously (Cold Spring Harbor Laboratory Press 2005).

*Chromatin immunoprecipitation (ChIP).* HEK293T and UROtsa cells were exposed to As_2_O_3_ for 3 or 72 hr, fixed with formaldehyde, and quenched with glycine. Primers used for ChIP are provided in Supplemental Material, Table S2. ChIP of samples was carried out as described previously (Nelson et al. 2006) and was followed by qRT-PCR. Enrichment of E2F1 in acetylated H3K9 promoters of *TP53, BAX, PUMA, C*-*MYC*, and *C*-*FOS* fragment precipitation was detected with anti-acetylated H3K9 and anti-E2F1 antibodies. Ten percent of the sample was set aside as input. C_t_ values were normalized according to the following equation: ΔC_t_(normalized ChIP) = {C_t_(ChIP) – [C_t_(input) – log_2_ (0.1)]}. The percent input was calculated using the following equation: percent input = 2^(–ΔC_t_[normalized ChIP])^.

## Results

**Figure 1:**
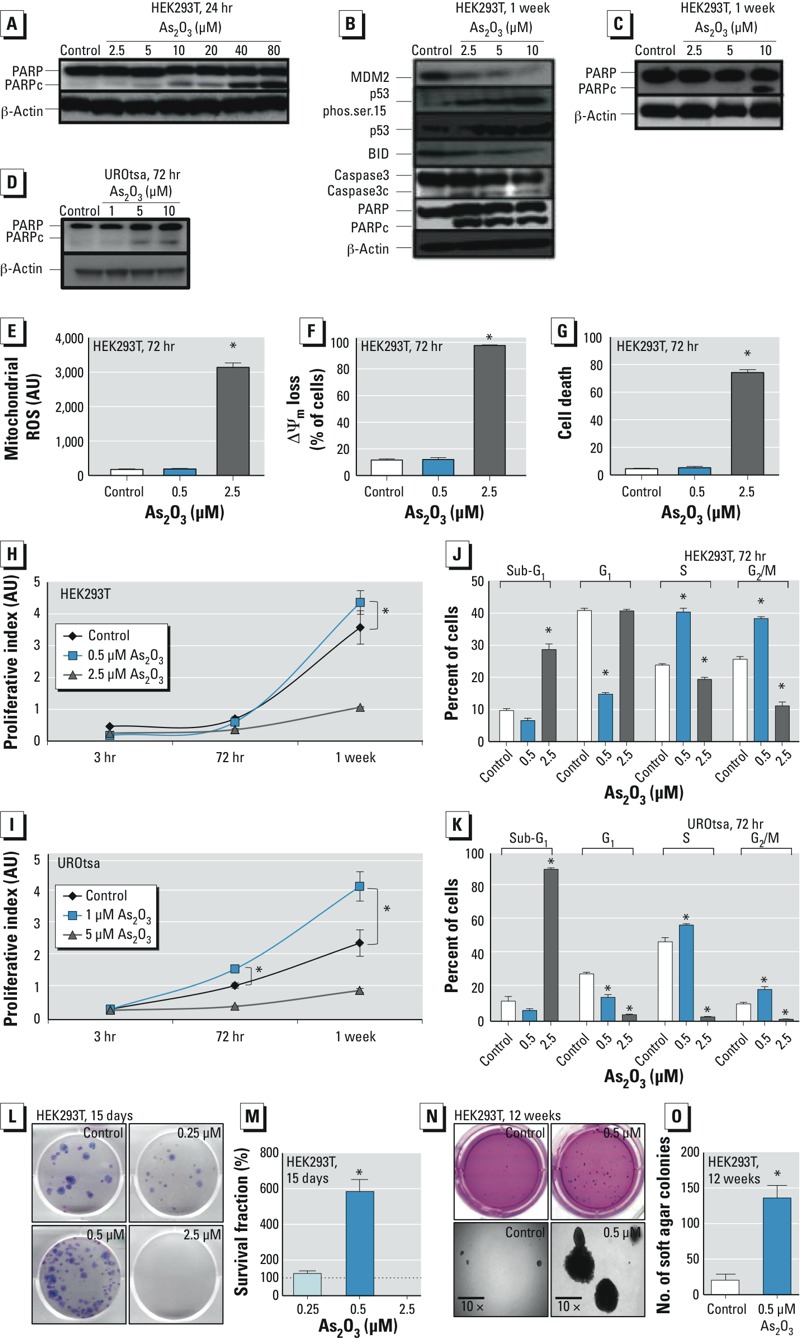
Identification of tolerated and toxic exposures of As_2_O_3_ (*A–D*) and cellular transformation assays in HEK293T and UROtsa cell lines after exposure to various doses of As_2_O_3_ (*E–O*). (*A–D*) Representative immunoblots showing PARP cleavage (PARPc) after 24‑hr exposure (*A*); MDM2, p53 activation, caspase-3 cleavage (Caspase3c), and apoptotic relay signals [phosphorylation at serine 15 (phos.ser.15), BID, and PARPc] after 1-week exposure (*B*); PARP cleavage after 1-week exposure (*C*); and PARP cleavage (*D*) after 72-hr exposure. (*E–G*) Quantification of flow cytometry results (presented as arbitrary units; AU) for mitochondrial ROS (*E*), mitochondrial membrane depolarization (∆Ψ_m_) (*F*), and cell death indicated by propidium iodide–bound DNA (*G*) after 72-hr exposure to 0.5 or 2.5 μM As_2_O_3_. (*H*,*I*) Quantification of WST-1 proliferation assay conducted to determine the proliferative index of HEK293T cells treated with 0.5 μM or 2.5 μM As_2_O_3_ (*H*) and UROtsa cells treated with 1 μM or 5 μM As_2_O_3_ (*H*) for 3 hr, 72 hr, or 168 hr (1 week). (*J*,*K*) Cell-cycle analysis of HEK293T cells treated with 0.5 μM or 2.5 μM As_2_O_3_ (*J*) and UROtsa cells treated with 1 μM or 5 μM As_2_O_3_ for 72 hr, determined by propidium iodide staining followed by flow cytometric analysis. (*L*) Representative images showing the number and approximate size of colonies of HEK293T cells after treatment with 0.5 μM or 2.5 μM As_2_O_3_ for 15 days, as determined in the clonogenic assay. (*M*) Quantification of the clonogenic assay of HEK293T cells after treatment with 0.5 μM or 2.5 μM As_2_O_3_ for 15 days; the survival fraction represents triplicate counting of cells. (*N*,*O*) Representative phase-contrast images (10×; *N*) and the number of colonies (*O*) of HEK293T cells grown in soft agar and treated with 0.5 μM As_2_O_3_ for 12 weeks. Values presented are mean ± SD. *p < 0.05 compared with the untreated control (*n* = 3), calculated by two-tailed Student’s *t*‑test.

*Identification of tolerated and toxic As_2_O_3_ exposures.* We used two cells lines in this study. HEK293T cells were used because they form a minimal number of colonies under anchorage-independent conditions, and they have good tolerance to transfection and a good toxicity response to As_2_O_3_. UROtsa cells were used because they exhibit normal characteristics with no colony formation in soft agar and are ideal for studying environmental insult to the human urothelium as well as bladder carcinogenesis (Rossi et al. 2001). To ensure characterization of the appropriate epigenetic context, we used the apoptotic end point of PARP cleavage to identify tolerated and toxic exposures of As_2_O_3_ in both cell lines. PARP cleavage was observed from 10 μM to 80 μM As_2_O_3_ for cells treated for 24 hr, and for 2.5 μM As_2_O_3_ for cells treated for 1 week for HEK293T cells (Figure 1A,B). Exposures of 2.5–10 μM As_2_O_3_ demonstrated cytotoxicity after 1 week, as assessed by caspase-3 cleavage, truncation of the pro-apoptotic factor BID, p53 stabilization, and activation by phosphorylation at serine 15 (Figure 1B). No PARP cleavage was observed after a 1-week exposure to 0.5 μM As_2_O_3_, suggesting that this exposure was tolerated (Figure 1C). UROtsa cells treated for 72 hr with 1 μM As_2_O_3_ showed no PARP cleavage, whereas higher exposures of 5 μM–10 μM As_2_O_3_ resulted in clear PARP cleavage (Figure 1D). This allowed us to define a tolerated exposure as 1 μM As_2_O_3_ and a toxic exposure as 5 μM As_2_O_3_ for the UROtsa cell line.

HEK293T cells treated for 72 hr with 0.5 μM As_2_O_3_ showed no discernable difference compared with the untreated control group in the generation of mitochondrial ROS or Δψ_m_ depolarization (Figure 1E,F). In cells treated with 0.5 μM As_2_O_3_, cell death was similar to that of controls, as quantified by propidium iodide staining (Figure 1G). In contrast, treatment with 2.5 μM As_2_O_3_ resulted in a significant increase in mitochondrial ROS, loss of Δψ_m_, and cell death. These analyses allowed us to define tolerated exposure as 0.5 μM As_2_O_3_ and toxic exposure as 2.5 μM As_2_O_3_ in the HEK293T cell line for exposures ≥ 72 hr.

*Cellular proliferation, survival, and anchorage-independent growth in response to a tolerated As_2_O_3_ exposure*. HEK293T cells exposed for 1 week to 0.5 μM As_2_O_3_ exhibited a significantly increased elevation in cellular proliferation (Figure 1H). We also observed a significant proliferative elevation in UROtsa cells exposed to 1 μM As_2_O_3_ for 72 hr or 1 week (Figure 1I). Cell cycle disruption was observed in both cell lines after 72-hr tolerated As_2_O_3_ exposure, with an increased percentage of cells passing the G_1_/S and G_2_/M checkpoints (Figure 1J,K). The clonogenic assay in HEK293T cells identified positive proliferation and cellular survival after treatment with 0.5 μM As_2_O_3_ for 15 days (Figure 1L,M). A lower dose of 0.25 μM As_2_O_3_, although tolerated, was unable to stimulate positive proliferation. Three-dimensional spheroid colonies grown in soft agar were larger and significantly increased in number in cells treated for 12 week with 0.5 μM As_2_O_3_ compared with untreated control cells (Figure 1N,O).

**Figure 2:**
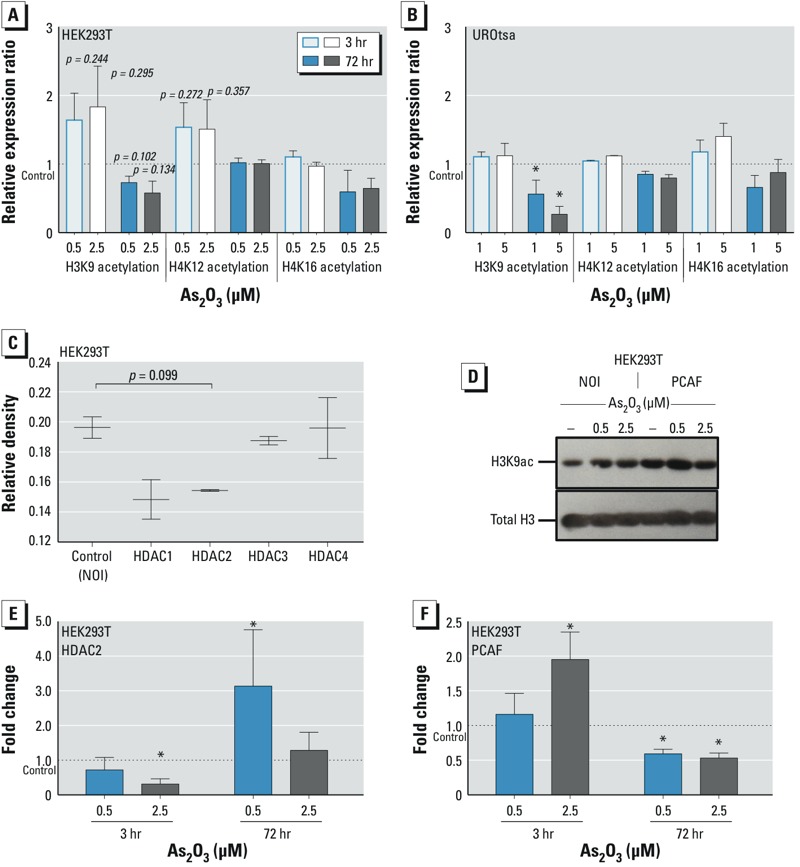
Histone acetylation analysis of HEK293T and UROtsa cells, identification of H3K9 acetylation control, and mRNA expression analysis of *HDAC2* and *PCAF* in cells treated with As_2_O_3_ for 3 or 72 hr. (*A*,*B*) Histone acetylation (mean ± SD) of H3K9, H4K12, and H4K16 in HEK293T (*A*) and UROtsa (*B*) cells, analyzed by immunoblotting and densitometric analysis (see “Materials and Methods” for details). (*C*) H3K9 acetylation status analyzed by immunoblotting after transfection of HEK293T cells with a no-insert control (NOI), HDAC1, HDAC2, HDAC3, or HDAC4, followed by histone extraction to examine the relative enzymatic effect of these HDACs on this residue; blots were quantified by densitometric analysis and values shown represent three independent experiments. (*D*) Representative immunoblot showing H3K9 acetylation (H3K9ac) status after transfection of HEK293T cells with a NOI or PCAF followed by a 3-hr exposure to 0.5 μM or 2.5 μM As_2_O_3_. (*E*,*F*) Relative mRNA expression ratios of *HDAC2 *(*E*) and PCAF* *(*F*), expressed as fold change for 3 and 72 hr. Values presented are mean ± SD. *p < 0.05 compared with the untreated control [*n* = 3 (*B,E*); *n* = 4 (*F* at 3 hr)] calculated by two-tailed Student’s *t*‑test.

*H3K9 and H4K12 acetylation and* HDAC2 *and* PCAF *mRNA expression profiles in response to As_2_O_3_*_._ Two time points were used to identify specific arsenic-induced histone acetylation changes and to evaluate the plasticity of such events in both the HEK293T and UROtsa cell lines. After 3-hr exposure to 0.5 μM or 2.5 μM As_2_O_3_, we observed an elevation of H3K9 and H4K12 acetylation in HEK293T cells (Figure 2A), but no change in H4K16 acetylation. In UROtsa cells, we observed a small, but not significant, increase in H3K9 and H4K16 acetylation after a 3-hr exposure to 1 μM or 5 μM As_2_O_3_ (Figure 2B). Significant H3K9 hypoacetylation was observed in UROtsa cells after a 72-hr exposure to both tolerated (1 μM) and toxic (5 μM) exposures. We observed a similar, but not significant, trend in HEK293T cells, in which treatment with 0.5 μM or 2.5 μM As_2_O_3_ resulted in hypoacetylation of H3K9 after 72 hr (Figure 2A).

Transient transfection followed by immunoblotting identified HDAC2 and PCAF as the key enzymes involved in controlling H3K9 acetylation status (Figure 2C,D). Transfection with *PCAF* elevated H3K9 acetylation relative to the control, and treatment with 0.5 μM As_2_O_3_ for 3 hr synergized this effect with enhanced acetylation of H3K9 (Figure 2D).

Using qRT-PCR, we measured dose-specific expression of *HDAC2*. After 3 hr, when both As_2_O_3_ doses are tolerated, *HDAC2* expression was significantly suppressed only at the 2.5 μM dose (Figure 2E). In contrast, after 72 hr of exposure, the *HDAC2* expression level was significantly elevated only at the tolerated 0.5-μM As_2_O_3_ dose, leaving nominal expression at 2.5 μM As_2_O_3_. Analysis of *PCAF* expression at the 3-hr time point revealed a significant increase in *PCAF* expression; however, at the 72-hr exposure, *PCAF* expression was suppressed at both tolerated and toxic doses (Figure 2F).

*Effects of E2F1 binding and H3K9 acetylation at selected gene promoters in response to As_2_O_3_*_._ To assess the relationship between histone acetylation and the transcriptional regulation of apoptotic genes and proto-oncogenes, we focused on the transcription factor E2F1, which has been shown to influence decisions between cellular proliferation and cell death (Chen et al. 1985; Hallstrom et al. 2008). E2F1 and H3K9 acetylation interactions at a number of gene promoters including the tumor suppressor *p53*, apoptotic genes *Bax* and *Puma*, and proto-oncogenes *Myc* and *Fos* were assessed by the ChIP assay and qRT-PCR.

**Figure 3:**
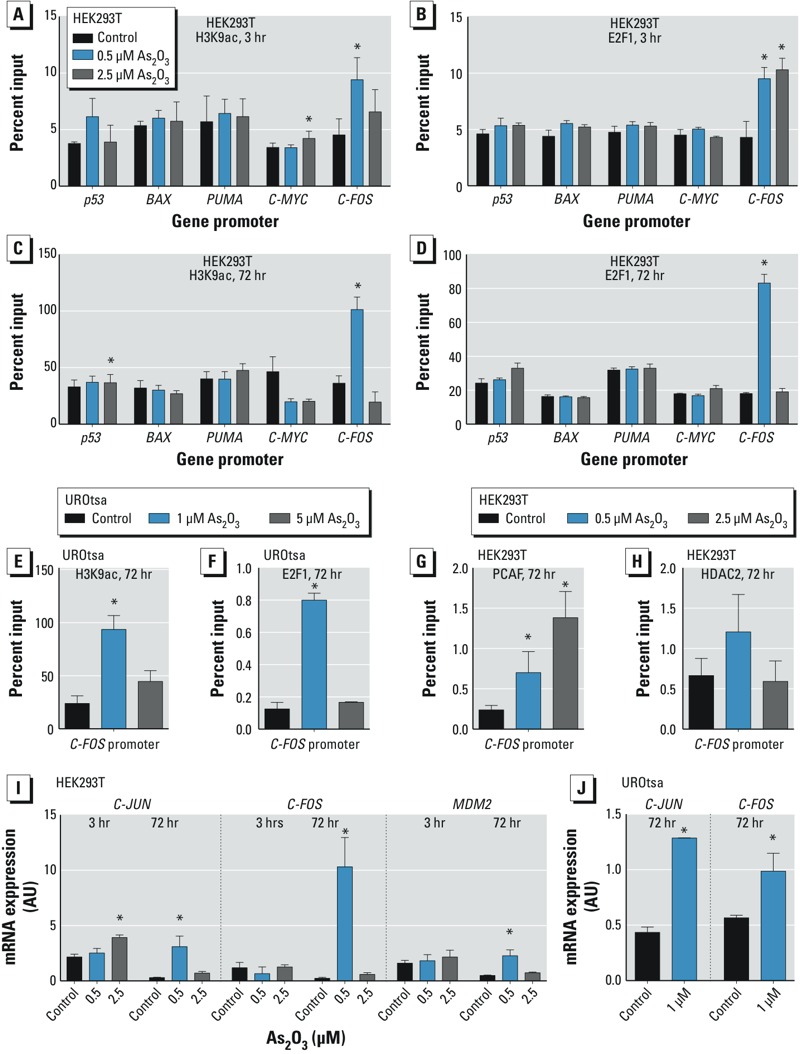
Tolerated As_2_O_3_ exposure induces the expression of *JUN*, *FOS*, and *MDM2* and leads to the recruitment of E2F1 at the FOS promoter. (*A*–*D*) Chromatin immunoprecipitated with anti-acetylated H3K9 (H3K9ac; *A,C*) or anti-E2F1 (*B,D*) antibodies after exposure of HEK293T cells to 0.5 μM or 2.5 μM As_2_O_3_ for 3 or 72 hr. (*E*,*F*) Chromatin immunoprecipitated with H3K9ac (*E*) and anti‑E2F1 (*F*) antibodies after exposure of UROtsa cells to 1 μM or 5 μM As_2_O_3_ for 72 hr. (*G*,*H*) Chromatin immunoprecipitated with anti-PCAF (*G*) and anti-HDAC2 (*H*) antibodies for 72 hr. Precipitated chromatin was subjected to qRT-PCR promoter analysis. (*I*) Normalized expression ratios for *JUN*, *FOS*, and *MDM2* mRNA; total RNA was isolated from HEK293T cells treated 3 or 72 hr with 0.5 μM or 2.5 μM As_2_O_3_ treated for use in qRT-PCR analysis. (*J*) Normalized expression ratios for *JUN* and *FOS* mRNA from UROtsa cells treated with 1 μM As_2_O_3_ for 72 hr. Values presented are mean ± SD. *p < 0.05 compared with the untreated control, calculated by two-tailed Student’s *t*-test (*n* = 3).

HEK293T cells were exposed to As_2_O_3_ for 3 or 72 hr. After 3 hr of exposure to 0.5 μM As_2_O_3_, there was a significant enrichment of the *FOS* promoter fragment after precipitation with an anti-acetylated H3K9 antibody (Figure 3A). This suggests an increase in H3K9 acetylation at the *FOS* promoter. Using the same duration of exposure and an anti-E2F1 antibody, we observed enrichment at the *FOS* promoter at both 0.5 μM and 2.5 μM As_2_O_3_ treatments, suggesting increased binding of E2F1 to the *FOS* promoter (Figure 3B). Dose-specific responses were observed after 72 hr exposure: With the use of both E2F1 and H3K9 acetylated antibodies, significantly elevated binding to the FOS promoter occurred specifically at the tolerated 0.5-μM As_2_O_3_ dose (Figure 3C,D). The same dose-specific response was validated in the UROtsa cell line, where 72-hr exposure to tolerated 1 μM As_2_O_3_ led to a significantly elevated binding of acetylated H3K9 and E2F1 at the *FOS* promoter fragment (Figure 3E,F).

We also examined the relative enrichment of PCAF and HDAC2 at the *FOS* promoter after a 72-hr exposure of HEK293T cells to determine whether these enzymes are involved in the maintenance of H3K9 acetylation (Figure 3G,H). There was increased binding of PCAF at the *FOS* promoter at the tolerated 0.5-μM dose and at the toxic 2.5-μM As_2_O_3_ dose. There was no significant difference in the HDAC2 binding at either exposure.

*Effects of As_2_O_3_ on* JUN, MDM2, *and* FOS *expression*. The effect of arsenic on the expression of the proto-oncogenes *JUN*, *FOS*, and *MDM2* was measured by qRT-PCR. The overexpression of these genes is synonymous with cancer stem cell expansion, p53 regulation, and proliferation (Ito et al. 2002; Jiao et al. 2010).

The changes in expression observed after 3 hr of exposure were subtle with a significant increase only in *JUN* expression with the 2.5-μM As_2_O_3_ treatment (Figure 3I). After 72 hr of exposure, the differences were more pronounced between the tolerated and toxic exposures. This is highlighted by the expression of *FOS*, which was highly elevated at the 0.5-μM As_2_O_3_ dose. This increase was dose specific: There was no significant elevation in cells treated with the toxic 2.5-μM As_2_O_3_ dose. We observed a similar expression pattern for *JUN*. A significantly elevated expression of the *FOS* and *JUN* mRNA was found in the UROtsa cell line after treatment with tolerated 1 μM As_2_O_3_ for 72 hr (Figure 3J). The expression of *MDM2* mRNA was significantly increased at the tolerated 0.5-μM As_2_O_3_ exposure in the HEK293T cell line.

**Figure 4:**
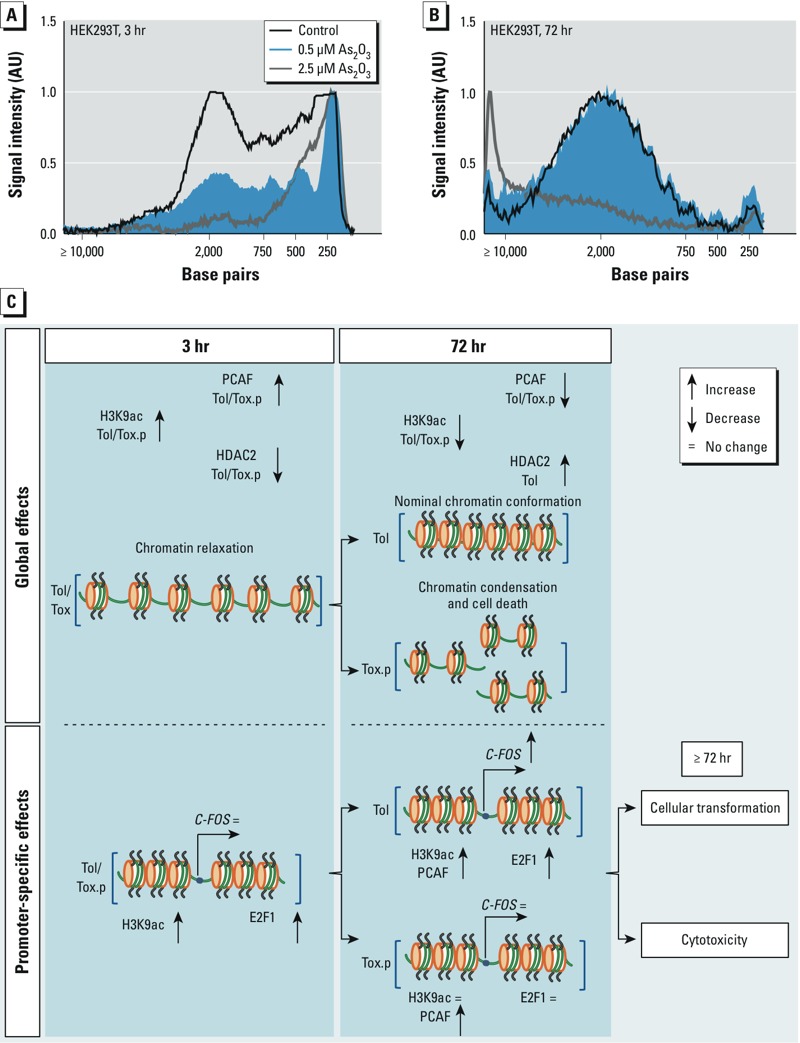
Tolerated and toxic As_2_O_3_ exposure alters higher-order chromatin conformation (*A*,*B*), and schematic of global and promoter-specific mechanisms in As_2_O_3_-induced cellular transformation (*C*). (*A*,*B*) MNase sensitivity assay was used to assess the relative levels of chromatin relaxation between 0.5 μM and 2.5 μM doses of As_2_O_3_ at 3 and 72 hr. (*C*) Schematic outlining HDAC2‑ and PCAF-mediated global acetylation changes in H3K9 followed by subsequent E2F1 recruitment to the *FOS* promoter. Tolerated and toxic doses led to separated mechanistic outcomes after 72 hr of exposure: a toxic dose led to cytotoxicity and chromatin condensation, whereas tolerated exposure resulted in continued *FOS* expression. Abbreviations: H3K9ac, acetylated H3K9; Tol, tolerated dose (0.5 μM As_2_O_3_); Tox.p, toxic pathway (2.5 μM As_2_O_3_ at 3 hr); Tox, toxic (2.5 μM As_2_O_3_ at ≥ 72 hr).

*Differential effects of tolerated and toxic As_2_O_3_ exposures on global chromatin conformation*. We used an MNase sensitivity assay to assess global chromatin conformation. Rapid relaxation occurred after 3 hr with 0.5-μM and 2.5-μM As_2_O_3_ treatment, and the digestion pattern was positively skewed toward the 250–500 base pair size range, relative to the 2,000 base pair size (Figure 4A). Treatment with 2.5 μM As_2_O_3_ led to greater chromatin relaxation than did 0.5 μM As_2_O_3_ after 3 hr. After 72 hr, this relaxation was reversed where the toxic 2.5-μM As_2_O_3_ treatment led to chromatin condensation (Figure 4B). At 72 hr, the tolerated 0.5-μM As_2_O_3_ exposure has the same digestion pattern as the control, with the majority of the DNA in the 2,000 base pair–size range, with a small elevation in the 250 base pair range compared with the control.

## Discussion

In this study we examined an *in vitro* framework to identify a tolerated dosage of As_2_O_3_ that induces cellular transformation as an important prerequisite to epigenetic characterization. To our knowledge, this initial dosage determination with knowledge of cellular outcome followed by the measurement of epigenetic parameters is undocumented. Multiple cellular transformation indicators ensured selection of the correct dosage and duration of exposure. In the absence of arsenic, cells showed no signs of deterioration or apoptosis, indicating that the culture conditions used were well tolerated. Cell line–specific tolerated and toxic dosages were determined. Both 0.5 μM and 2.5 μM As_2_O_3_ were tolerated at 3 hr in HEK293T cells; at 72 hr the 0.5 μM dose remained tolerated, but 2.5 μM As_2_O_3_ was toxic. In UROtsa cells, 1 μM was tolerated but 5 μM As_2_O_3_ was toxic at 72 hr. Both toxic exposures stimulated apoptosis after a 72-hr exposure. In contrast, tolerated exposures were observed to potentiate transformative properties, including positive proliferation and a redistributed cell cycle to pass through the G_1_/S and G_2_/M checkpoints in both cell lines. Increased cellular survival and an elevated number of colonies grown under anchorage-independent conditions were observed in HEK293T cells after a tolerated exposure.

These findings further our understanding of the paradoxical effects of As_2_O_3_ as an anticancer agent and carcinogen. Although it may be effective for some malignancies, As_2_O_3_ treatment may not be appropriate for kidney and urothelial cancers, because we observed that tolerated exposures may lead to cellular transformation rather than cell death. The dichotomy of arsenicals as a potent cancer treatment and a carcinogenic compound is still under intense investigation. Additional research into these effects has attempted to compare and contrast the toxicity of multiple arsenicals. Treatment of human lung adenocarcinoma A549 cells with 15 μM sodium arsenite (NaAsO_2_) resulted in an increase of cells passing the G_1_/S checkpoint, in contrast to 15 μM As_2_O_3_, which led to a significant decrease, suggesting that sodium arsenite is potentially more carcinogenic (Jiang et al. 2013). Doses lower than 5 μM were not a focus of that study, and the exposure duration was 24 hr, leading to toxic cellular outcomes. In another study, Zhou et al. (2014) compared the toxicity of As_2_O_3,_ NaAsO_2_, and monomethylarsonous acid (MMA^III^) in the context of zinc finger peptides and found that MMA^III^ changed the conformation and displaced zinc in C2H2, C3H1, and C4 zinc finger proteins isolated from human keratinocyte HaCaT cells. These studies demonstrate toxicity and carcinogenicity variations that are dependent on the species of arsenic, and suggest that the dosage and duration of exposure are also significant, as we found in the present study.

Using tolerated and toxic dosages, we observed temporally sensitive changes in global H3K9, H4K12, and H4K16 acetylation as a result of tolerated As_2_O_3_ exposure in both cell lines. Prior research has identified a mixture of promoter-specific and global histone acetylation changes. Altered histone H3 acetylation patterns in the *DBC1*, *FAM83A*, *ZSCAN12*, and *C1QTNF6* gene promoters have been reported as a result of exposure to an intermediate of arsenic catabolism, MMA^III^ (Jensen et al. 2008). Our observed reduction of H4K16 acetylation, although not significant, is in agreement with previous research in which a decreasing H4K16 acetylation profile after chronic exposure to 3 μM As_2_O_3_ and 1 μM MMA^III^ was believed to protect cells from arsenic toxicity (Jo et al. 2009). Urinary arsenic concentration and H3K9 acetylation in peripheral blood mononuclear cells are inversely correlated, which is concomitant with our observed hypoacetylation of H3K9 at both tolerated and toxic exposures in both cell lines at 72 hr (Chervona et al. 2012). Our data suggest that hypoacetylation of H3K9 and H4K16 may be involved in the intermediary steps toward cellular transformation. These end points alone, however, did not give clear mechanistic separation between tolerated and toxic outcomes because both exposures induced hypoacetylation.

We examined the association between hypoacetylation and cellular transformation by identifying the enzymes HDAC2 and PCAF as regulators of H3K9 acetylation in HEK293T cells. Subsequent investigation into *HDAC2* and *PCAF* mRNA levels by qRT-PCR demonstrated that the initial increase in global H3K9 acetylation was potentially caused by an adjustment in the *HDAC2* to *PCAF* expression ratio. The direction of this imbalance was inverted at the 72-hr time point, where, at the tolerated exposure of 0.5 μM As_2_O_3_, *HDAC2* expression was significantly elevated and *PCAF* expression was significantly reduced, leading to global hypoacetylation of the H3K9 residue at the 72-hr time point in HEK293T cells.

Although aberrant PCAF and HDAC2 expression in the context of arsenic-induced carcinogenicity has not yet been documented, both PCAF and HDAC2 have been implicated in cancer. The role of PCAF is not definitive in carcinogenesis, but there is growing consensus that PCAF has tumor-suppressive properties (Zheng et al. 2013). HDAC2 is also believed to interact with cell cycle components, where siRNA-mediated silencing of HDAC2 inhibits progression through the G_1_/S checkpoint in hepatocarcinoma cells (Noh et al. 2011). This is particularly significant because, in our study, tolerated As_2_O_3_ exposure led to a redistributed cell cycle to favor cellular proliferation by passing both G_1_/S and G_2_/M checkpoints in both cell lines.

To assess the significance of global H3K9 hypoacetylation in the transcriptional control of proto-oncogenes and apoptotic genes, we used chromatin immunoprecipitation to focus on the transcription factor E2F1 because of its dual role in proliferation and apoptosis (Korah et al. 2012). We investigated the binding of E2F1 at a number of promoters and identified the co-occurrence of promoter-specific H3K9 acetylation and E2F1 at the *FOS* promoter, a well-established proto-oncogene in HEK293T and UROtsa cells. At the 72-hr time point, this interaction was significantly strengthened at the tolerated concentration. E2F1 has been previously shown to interact in the promoter region of *FOS* and the retinoblastoma control element (RCE) and regulate the transcription of the pRb protein, an important regulatory protein in balancing cellular proliferation and apoptosis (Mimaki et al. 2005). In the present study, we found that at 3 hr, H3K9 was hyperacetylated at the *FOS* promoter, similar to the global acetylation profile. This localized acetylation was able to stimulate significant E2F1 binding at both tolerated and toxic exposures. This adjusted after 72 hr of exposure because H3K9 acetylation was maintained at the *FOS* promoter specifically at the tolerated treatment in both cell lines. Although global H3K9 acetylation was reduced at this exposure, E2F1 transcription factor binding was still secure at the *FOS* promoter leading to transcription. It has been previously documented that E2F1 is able to recruit other histone acetyltransferases such as GCN5 to acetylate localized H3K9 residues (Guo et al. 2011). We predict that a potentially similar mechanism is involved because we identified elevated binding of PCAF at the *FOS* promoter in concert with E2F1 after a tolerated treatment for 72 hr. This allowed for the maintained H3K9 acetylation at the promoter. Although we observed a similar increase in PCAF binding at the toxic exposure, this did not occur with E2F1 binding at the promoter, leaving *FOS* expression and H3K9 acetylation nominal. Furthermore, combined transfection with PCAF and treatment at the toxic dosage led to inhibited acetylation activity on the H3K9 substrate compared with transfected untreated controls.

Our data show how arsenic-induced changes in global histone acetylation may not always be reflected at the promoter-specific level. Rather, early perturbations in global histone acetylation may lead to transcription factor binding and promoter-specific effects. In our proposed mechanism, arsenic exposure led to an adjustment in HDAC2 and PCAF expression and hyperacetylation of the H3K9 residue within 3 hr (Figure 4C). This may allow for the early recruitment of E2F1, which after 72 hr resulted in PCAF-mediated H3K9 hyperacetylation at the promoter and sustained transcription. This promoter-specific effect occurred against a background of global H3K9 hypoacetylation. This understanding of arsenic-induced global acetylation followed by focused profiling at a specific gene promoter loci is a significant step in understanding arsenic-induced carcinogenesis from perturbations in histone posttranslational status.

Transcriptional regulation and higher-order chromatin conformation are intrinsically tethered by histone acetylation events that adjust the electrostatic properties of the histones (Shogren-Knaak 2006). We observed chromatin relaxation in a dose-dependent manner in addition to increases in H3K9 and H4K12 acetylation at the 3-hr time point in HEK293T cells. Extension of the exposure to 72 hr led to chromatin condensation in the toxic exposure but normalization of the chromatin architecture at the tolerated exposure in HEK293T cells. This demonstrates the reversibility of initial relaxation, and this co-occurred with the global hypoacetylation of H3K9 and normalization of H412 acetylation in HEK293T cells. Similar condensation was observed as a result of As_2_O_3_-initiated apoptosis in the treatment of hepatocarcinoma (Alarifi et al. 2013). This may explain why initial E2F1 binding at the toxic exposure was not maintained after a 72-hr exposure, because the chromatin was severely condensed and E2F1 binding became unfavorable at the *FOS* promoter. We also measured the mRNA levels of other proto-oncogenes *JUN* and *MDM2*, both of which after 72 hr exhibited significantly elevated expression at the tolerated As_2_O_3_ exposure, further contributing to cellular transformation.

## Conclusions

This study provides additional mechanistic detail to arsenic-induced cellular transformation beginning with the analysis of histone posttranslation modifications. We believe that global histone acetylation changes and promoter-specific impacts are not entirely congruent and that promoter-specific histone acetylation changes are more accurate in separating the opposing physiological outcomes of cellular transformation and toxicity. In summary, this research outlined a framework for investigating arsenic-induced carcinogenesis by using a clear dosage selection methodology. Tolerated As_2_O_3_ exposure led to early an H3K9 acetylation increase in HEK293T and UROtsa cells mediated by an imbalance in the intracellular HDAC2 to PCAF expression ratio as observed in HEK293T cells. This event allowed for the subsequent binding of E2F1 at the *FOS* promoter, which maintained promoter-localized H3K9 acetylation, against the global H3K9 hypoacetylation trend observed at the tolerated exposure in both cell lines. We also observed that short-term As_2_O_3_ induced chromatin relaxation in HEK293T cells and then a return to nominal levels for the tolerated concentration, in contrast to the toxic exposure, which led to clear chromatin condensation and apoptosis. The identification of more promoter targets regulated by histone acetylation as a result of tolerated As_2_O_3_ exposure may help to further our understanding of arsenic-induced cellular transformation.

## Supplemental Material

(163 KB) PDF
