# Supplementary material for: E2F1-Mediated FOS Induction in Arsenic Trioxide–Induced Cellular Transformation: Effects of Global H3K9 Hypoacetylation and Promoter-Specific Hyperacetylation in Vitro
Source: Environ Health Perspect. 2015 Jan 9;123(5):484–92. doi: 10.1289/ehp.1408302 (PMC4421767; doi:10.1289/ehp.1408302)
Supplement: (163 KB) PDF [file ehp.1408302.s001.508.pdf]

Environmental Health Perspectives doi:10.1289/ehp.1408302

Supplemental Material

E2F1-Mediated *FOS* Induction in Arsenic Trioxide–Induced Cellular Transformation: Effects of Global H3K9 Hypoacetylation and Promoter-Specific Hyperacetylation *in Vitro*

Sunniyat Rahman, Zjwan Housein, Aleksandra Dabrowska, Maria Dolores Mayán, Alan R. Boobis, and Nabil Hajji

Plasmids

Plasmids used were HDAC1 (Addgene # 13820), HDAC3 (Addgene # 13819) and HDAC4 (Addgene # 13821). HDAC2 was generously provided by Dr. Ito Kazuhiro (Imperial College London). PCAF, HMOF and TIP60 were kindly provided by Dr. Bertrand Joseph (Karolinska Institutet).

Antibodies

For histone acetylation analysis the following antibodies were used: anti-acetyl Histone H3-Lys 9, anti-acetyl Histone H4-Lys 12 and anti-acetyl Histone H4-Lys 16 (all from Millipore). For intracellular protein analysis the following antibodies were used: anti-PARP, Mdm2, p53 phosphorylated serine 15, p53, Bid, Caspase3 and ß-Actin (all from Cell Signalling).

Table S1. Primers used for qRT-PCR.

Target	Forward 5'-> 3'	Reverse 5'-> 3'
cFOS	TCGGGCTTCAACGCAGACTACG	AAGGAGTCTGCGGGTGAGTGGT
cJUN	AAGCGCATGAGGAACCGCATCG	TCACTTTTTCCTCCAGCCGGGC
MDM2	TTCCCAGCCTAGGTTTCAGA	AACACGGAGCTTGAGAGGAA
P53	GTGGTTTCAAGGCCAGATGT	GGCCCACTTCACCGTACTAA

Table S2. Primers used for ChIP.

**Target Promoter**	**Forward 5'-> 3'**	**Reverse 5' -> 3'**
P53	CAGTTGCAAACCAGACCTCA	GTGGAAGGAAATTTGCGTGT
BAX	GAGACACTCGCTCAGCTTCTT	TTCATCCAGGATCGAGCAG
PUMA	GGACAGTCGGACACACACAC	GTACATCCTCTGGGCTCTGC
C-MYC	TGGCGGGAAAAAGAACGGAG	GAAGCCGCTCCACATACAGT
C-FOS	CAGACTACGAGGCGTCATCC	AGTTGGTCTGTCTCCGCTTG
